# Prevalence and factors associated with long COVID in adults from Southern Brazil: findings from the PAMPA cohort

**DOI:** 10.1590/0102-311XEN098023

**Published:** 2023-12-11

**Authors:** Natan Feter, Eduardo Lucia Caputo, Jayne Santos Leite, Felipe Mendes Delpino, Luísa Silveira da Silva, Yohana Pereira Vieira, Isabel de Almeida Paz, Juliana Quadros Santos Rocha, Carine Nascimento da Silva, Natália Schröeder, Marcelo Cozzensa da Silva, Airton José Rombaldi

**Affiliations:** 1 Universidade Federal do Rio Grande do Sul, Porto Alegre, Brasil.; 2 Universidade Federal de Pelotas, Pelotas, Brasil.; 3 Universidade Federal do Rio Grande, Rio Grande, Brasil.

**Keywords:** Long COVID, Vaccination, Cohort Studies, COVID Longa, Vacinação, Estudos de Coortes, COVID Largo, Vacunación, Estudios de Cohortes

## Abstract

Most COVID-19 survivors have reported experiencing persistent symptoms after the infection - these types of cases are known as long COVID. Since Brazil was an epicenter of the COVID-19 pandemic, a high burden of long COVID is expected. This study aimed to identify the prevalence and factors associated with long COVID in adults in Southern Brazil, analyzing data from the PAMPA cohort. Participants filled out a self-reported online questionnaire in June 2022. This study only included subjects who tested positive for COVID-19. Long COVID was defined by any symptoms that persisted for at least three months after the SARS-CoV-2 infection. Poisson’s regression models with robust variance were used to identify factors associated with long COVID; and results were reported as prevalence ratios (PR) and respective 95% confidence intervals (95%CI). A total of 1,001 participants (77.4% women, mean age [SD] = 38.3 [11.9] years) were analyzed. The prevalence of long COVID among these patients was 77.4% (95%CI: 74.7; 79.9). The likelihood of long COVID was higher in unvaccinated participants (PR = 1.23, 95%CI: 1.06; 1.42), in those with chronic conditions (PR = 1.13, 95%CI: 1.04; 1.24), and in those who were hospitalized due to the COVID-19 infection (PR = 1.24, 95%CI: 1.16; 1.32). This prevalence was also higher in women (PR = 1.21, 95%CI: 1.09; 1.33) than in men. Physical activity was associated with a reduced likelihood of fatigue, neurological complications, coughing, and headaches as persistent symptoms after a COVID-19 infection. It was found that three out of four adults in Southern Brazil experienced long COVID. Public policies aiming to reduce the burden of long COVID must be prioritized, especially in groups that are at higher risk of developing this harmful condition.

## Resumo

A maior parte dos sobreviventes da COVID-19 relatou sintomas persistentes após a infecção, também conhecida como COVID longa. O Brasil foi um epicentro da pandemia de COVID-19, logo, espera-se uma alta carga de COVID longa. Este estudo teve como objetivo identificar a prevalência e os fatores associados à COVID longa em adultos no Sul do Brasil. Foram analisados dados da coorte PAMPA. Os participantes preencheram um questionário *online* autoaplicável em junho de 2022. Foram incluídos apenas os participantes que testaram positivo para COVID-19. A COVID longa foi definida como qualquer sintoma que persistiu por pelo menos três meses após a infecção do SARS-CoV-2. Os modelos de regressão de Poisson com variância robusta foram usados para identificar fatores associados à COVID longa; os resultados foram relatados como razão de prevalência (RP) e seus respectivos intervalos de 95% de confiança (IC95%). Ao todo, 1.001 participantes (77,4% mulheres, idade média [DP] = 38,3 [11,9] anos) foram analisados. A prevalência da COVID longa foi de 77,4% (IC95%: 74,7; 79,9). A probabilidade da COVID longa foi maior em participantes não vacinados (RP = 1,23, IC95%: 1,06; 1,42), pessoas com condições crônicas (RP = 1,13, IC95%: 1,04; 1,24), e pacientes hospitalizados devido à infecção por COVID-19 (RP = 1,24, IC95%: 1,16; 1,32) em comparação com as contrapartes. A prevalência foi maior em mulheres (RP = 1,21, IC95%: 1,09; 1,33) do que em homens. A atividade física foi associada à probabilidade reduzida de fadiga, complicações neurológicas, tosse e dor de cabeça como sintomas persistentes após a infecção por COVID-19. Três em cada quatro adultos no Sul do Brasil tiveram COVID longa. Políticas públicas que visem reduzir a carga da covid longa devem ser priorizadas, especialmente nos grupos de maior risco desta condição.

## Resumen

La mayoría de los supervivientes de la COVID-19 relató síntomas persistentes tras la infección, también conocida como COVID largo. Brasil fue un epicentro de la pandemia de COVID-19, así, se espera una alta carga de COVID largo. El objetivo de este estudio fue identificar la prevalencia y los factores asociados con la COVID largo en adultos en el Sur de Brasil. Se analizaron datos de la cohorte PAMPA. Los participantes rellenaron un cuestionario en línea autoadministrado en junio de 2022. Solo se incluyeron los participantes que dieron positivo para COVID-19. La COVID largo fue definida como cualquier síntoma que persistió durante al menos tres meses tras la infección del SARS-CoV-2. Se utilizaron los modelos de regresión de Poisson con varianza robusta para identificar factores asociados con el COVID largo; se relataron los resultados como razón de prevalencia (RP) y sus respectivos intervalos de 95% confianza de 95% (IC95%). En total, se analizaron 1.001 participantes (el 77,4% mujeres, edad media [DP] = 38,3 [11,9] años). La prevalencia del COVID largo fue del 77,4% (IC95%: 74,7; 79,9). La probabilidad del COVID largo fue más alta en participantes no vacunados (RP = 1,23, IC95%: 1,06; 1,42), personas con condiciones crónicas (RP = 1,13, IC95%: 1,04; 1,24) y pacientes hospitalizados debido a la infección por COVID-19 (RP = 1,24, IC95%: 1,16; 1,32) en comparación con sus contrapartes. La prevalencia fue más alta en mujeres (RP = 1,21, IC95%: 1,09; 1,33) que en hombres. La actividad física se asoció con la probabilidad reducida de fatiga, complicaciones neurológicas, tos y dolor de cabeza como síntomas persistentes tras la infección por COVID-19. Tres de cada cuatro adultos en el Sur de Brasil han tenido COVID largo. Se deben priorizar las políticas públicas destinadas a reducir la carga del COVID largo, sobre todo en los grupos de mayor riesgo para esta condición.

## Introduction

Long COVID is a new and emerging clinical phenomenon observed in many individuals recovering from COVID-19 ^1^ ^,^ ^2^. This condition is characterized by symptoms and complications that persist for at least 12 weeks after the initial COVID-19 infection is resolved ^2^ ^,^ ^3^. The symptoms of long COVID can vary widely and may include fatigue, shortness of breath, chest pain, joint pain, brain fog, and other neurological and psychological manifestations ^4^. Therefore, identifying factors associated with long COVID is an emerging priority in public health in order to reduce the burden of this condition.

Estimates of the prevalence of long COVID vary in different studies: some previous meta-analyses estimate that up to 80% of COVID-19 survivors developed persistent symptoms after the infection ^1^ ^,^ ^4^. The prevalence of this condition has been shown to be higher in adults than in children or adolescents ^5^. Brazil was one of the epicenters of the COVID-19 pandemic, with 37.5 million confirmed cases of the disease until April 2023 ^6^. However, the official figures probably underestimate the true prevalence of long COVID, mainly due to the limited access to testing and asymptomatic cases ^7^ ^,^ ^8^. Given the potentially long-lasting and debilitating nature of long COVID, there is an urgent need for greater research efforts to better understand this phenomenon and develop effective treatments and interventions for affected individuals. However, studies estimating the factors associated with long COVID in Brazil are scarce. Therefore, we aimed to assess the proportion of adults with long COVID in Southern Brazil and the factors associated with this chronic condition.

## Methods

### Study design

Data from the *Prospective Study about Mental and Physical Health* (PAMPA cohort) were analyzed. The PAMPA cohort is an ongoing, longitudinal study investigating the indirect and direct consequences of the COVID-19 pandemic in adults living in Rio Grande do Sul, southernmost Brazilian state. This study was approved by the Research Ethics Committee of the Higher School of Physical Education at Federal University of Pelotas (CAAE: 31906920.7.0000.5313). More details about study design and recruitment can be found elsewhere ^9^.

### Participant recruitment and sample

Adults (18 years old or over) living in the Rio Grande do Sul State were recruited for this study using four different approaches, aiming to reach participants from all state regions. Researchers spread the weblink of the questionnaire via (1) messages, sharing it with their personal and professional contacts over the state; (2) social media campaigns; (3) local media and state agencies; and (4) faculty and students ^9^. In this study, we used data from wave four (June and July 2022), which was the first data collection process with questions about persistent symptoms after SARS-CoV-2 infection. In total, 2,545 subjects took part in the wave four collection, and 1,001 reported getting positive results in PCR or rapid COVID-19 tests, which were included in this study.

### Long COVID

Participants who reported testing positive for COVID-19 received a list of symptoms (e.g., fatigue, coughing, headache) and were asked to report which ones they had experienced. For each symptom, subjects could choose one of the following options: (1) did not experience it, (2) experienced it for three months, (3) experienced it for six months, (4) experienced it for 12 months, (5) experienced it for 15 months, and (6) experienced it for 18 months or more. Based on this data, long COVID was defined as experiencing any COVID-related symptoms for at least three months. The experience of each persistent symptom, which will henceforward be referred to as long COVID symptom-specific, was also identified. Only symptoms with a prevalence ≥ 20% were considered for analysis purposes. Memory and concentration problems, irritability, depression, and anxiety were grouped under the term “neurological complications” in the analyses.

### Exposure

Demographic, behavioral, and clinical characteristics were analyzed using hierarchical models. Model 1 included demographic factors: sex (male, female); age (in years); educational level (high school or less, university degree or higher); family income (in minimum wages); race/ethnicity (white, black, Asian, mixed-race/*pardo*); and occupational status (unemployed, employed, student, employed and student, retired, financially supported by the Federal Government, or other). Model 2 included behavioral, nutritional, and clinical factors: smoking status (current smoker, former-smoker, never had a smoking habit), body mass index - BMI (normal, overweight, and obese), alcohol consumption (none, < 1 day/week, 1-2 days/week, 3-4 days/week, ≥ 5 days/week), physical activity (inactive, active), and chronic conditions (no, yes). Participants’ self-reported weight and height were assessed to calculate BMI, which was categorized as follows: normal (< 25kg/m^2^), overweight (≥ 25 and < 30kg/m^2^), and obese (≥ 30kg/m^2^), in accordance with the World Health Organization (WHO) ^10^. Model 3 included COVID-19-related factors: whether the participant was hospitalized due to COVID-19 infection and vaccination schedule (unvaccinated, one or two doses, three or more doses). During the survey period, adults who had taken three to four vaccine doses were considered to have a complete immunization, since the fourth dose, also known as the second boost, was only available for some age groups at that time.

### Statistical analysis

Descriptive data are presented as total and relative frequencies. Factors associated with general and specific long COVID symptoms were identified using robust Poisson’s regression models, which provide more reliable relative risk estimates than logistic regression when analyzing binary outcomes from cross-sectional studies ^11^. A hierarchical model of determination based on the conceptual framework was constructed for analysis purposes. This model allows quantifying the contribution of each level for experiencing long COVID symptoms. Confounder control was carried out for variables at the same level or at levels immediately superior to each other. The final multivariable model maintained variables with a p-value < 0.20. The associations between explanatory variables and outcomes were assessed using the Wald test, with a 5% significance threshold in univariable and multivariable analyses. The statistical package Stata version 14.2 (https://www.stata.com) was used for the analyses. Data are presented as prevalence ratio (PR) with their respective 95% confidence interval (95%CI).

## Results

We analyzed data from 1,001 participants of the PAMPA cohort. The initial sample included 2,545 adults, but those who did not test positive for COVID-19 were excluded. Three out of four adults participating in our study had long COVID (Table 1). The proportion of women and men with long COVID compared to the overall sex-specific sample was 81.2% and 66.4%, respectively. Participants with long COVID were also more likely have obesity, physically inactive, and have chronic conditions. In addition, the proportion of long COVID was lower among those with a higher monthly income.

**Table 1:** Sample characteristics. PAMPA cohort (N = 1,001).

Characteristics	Long COVID		p-value
	No (n = 226)	Yes (n = 775)	
	n (%)	n (%)	
Sex			< 0.001 *
Male	86 (38.1)	170 (21.9)	
Female	140 (61.9)	605 (78.1)	
Age (years) [mean (SD)]	37.8 (11.3)	38.4 (12.1)	0.537 **
Family income (minimum wages)			0.006
< 1	5 (2.3)	41 (5.4)	
1-2	22 (10.0)	110 (14.6)	
3-4	52 (23.7)	215 (28.5)	
> 4	140 (63.9)	388 (51.5)	
Race/Ethnicity			0.320 *
White	193 (85.4)	693 (89.4)	
Black	12 (5.3)	33 (4.3)	
Asian	0 (0.0)	1 (0.1)	
Mixed-race/*Pardo*	21 (9.3)	48 (6.2)	
Occupational status			0.330 *
Employed	136 (60.2)	402 (51.9)	
Unemployed	11 (4.9)	51 (6.6)	
Student	23 (10.2)	72 (9.3)	
Employed and student	45 (19.9)	187 (24.1)	
Retired	9 (4.0)	43 (5.5)	
Supported by the Federal Government	0 (0.0)	6 (0.8)	
Other	2 (0.9)	14 (1.8)	
Smoking			0.180 *
Smoker	9 (4.0)	57 (7.4)	
Former-smoker	25 (11.1)	90 (11.6)	
Never smoked	192 (85.0)	628 (81.0)	
BMI			0.004
Normal	104 (46.0)	339 (43.9)	
Overweight	87 (38.5)	237 (30.7)	
Obese	35 (15.5)	197 (25.5)	
Physical activity			0.014 *
Inactive	109 (48.2)	445 (57.5)	
Active	117 (51.8)	329 (42.5)	
Hospitalized due to COVID-19			0.002 *
No	225 (100.0)	743 (95.9)	
Yes	0 (0.0)	32 (4.1)	
COVID-19 vaccination (doses)			0.270
None	0 (0.0)	5 (0.6)	
1-2	28 (13.3)	126 (16.3)	
3-4	183 (86.7)	642 (83.1)	
Chronic condition			< 0.001 *
No	84 (37.2)	185 (23.9)	
Yes	142 (62.8)	590 (76.1)	

BMI: body mass index; SD: standard deviation.

* Chi-squared test;

** Linear trend test.

We investigated the sociodemographic, behavioral, and clinical factors associated with long COVID, as shown in Table 2. In Model 1, age was not associated with long COVID (p-value = 0.590), but the variables family income, race/ethnicity, and occupational status were significantly associated with it. However, these associations did not remain present when Models 2 and 3 were included in the analysis. Sex was the only sociodemographic factor that remained associated to long COVID in the fully adjusted model (PR = 1.19, 95%CI: 1.08; 1.31), as illustrated in Table 2. Smoking (p-value = 0.241), alcohol consumption (p-value = 0.393), and physical activity (p-value = 0.166) were not associated with long COVID. Obesity was associated with a higher probability of long COVID than normal BMI in Model 2 (PR = 1.09; 95%CI: 1.01; 1.18), but no significant association between these two variables was observed in the fully adjusted model (PR = 1.06, 95%CI: 0.98; 1.15). Participants who had not taken the vaccine against COVID-19 and those who received up to two doses were 23% (95%CI: 1.08; 1.41) and 8% (95%CI: 1.01; 1.18), respectively, more likely to have long COVID than those who took three to four doses. Finally, participants who underwent hospitalization due to COVID-19 infection (PR = 1.24, 95%CI: 1.16; 1.32) and those living with chronic conditions (PR = 1.13, 95%CI: 1.03; 1.24) had a higher likelihood of developing long COVID than their counterparts.

**Table 2:** Factors associated with the likelihood of long COVID in adults living in Southern Brazil (N = 1,001).

Characteristics	Model 1	p-value	Model 2	p-value	Model 3	p-value
	PR (95%CI)		PR (95%CI)		PR (95%CI)	
Sex		< 0.001		< 0.001		< 0.001
Male	Reference		Reference		Reference	
Female	1.19 (1.08; 1.31)		1.18 (1.07; 1.30)		1.19 (1.08; 1.31)	
Age (years)	1.00 (1.00; 1.01)	0.590				
Education		0.449				
High school or less	1.00					
University degree or higher	0.97 (0.89; 1.05)					
Family income (minimum wages)		0.001		0.003		0.148
< 1	Reference		Reference		Reference	
1-2	0.94 (0.83; 1.06)		0.94 (0.83; 1.06)		0.99 (0.87; 1.12)	
3-4	0.90 (0.80; 1.02)		0.90 (0.80; 1.02)		0.97 (0.85; 1.09)	
> 4	0.84 (0.75; 0.94)		0.85 (0.76; 0.96)		0.93 (0.83; 1.06)	
Race/Ethnicity		< 0.001		0.002		0.280
White	Reference		Reference		Reference	
Black	0.91 (0.77; 1.09)		0.91 (0.77; 1.09)		0.96 (0.82; 1.12)	
Asian	1.37 (1.25; 1.54)		1.25 (1.09; 1.42)		-	
Mixed-race/*Pardo*	0.87 (0.74; 1.01)		0.88 (0.75; 1.03)		0.89 (0.77; 1.03)	
Occupational status		< 0.001		0.024		0.367
Employed	Reference		Reference		Reference	
Unemployed	1.01 (0.87; 1.15)		0.99 (0.86; 1.13)		0.95 (0.83; 1.09)	
Student	0.99 (0.87; 1.12)		1.00 (0.88; 1.13)		0.98 (0.86; 1.10)	
Employed and student	1.05 (0.97; 1.14)		1.06 (0.98; 1.15)		1.06 (0.98; 1.15)	
Retired	1.10 (0.96; 1.25)		1.09 (0.96; 1.24)		1.06 (0.93; 1.21)	
Supported by Federal Government	1.24 (1.13; 1.36)		1.21 (1.09; 1.33)		1.10 (0.98; 1.24)	
Other	1.09 (0.89; 1.33)		1.04 (0.85; 1.27)		0.97 (0.78; 1.19)	
Smoking				0.241		
Smoker			Reference			
Former-smoker			0.92 (0.82; 1.29)			
Never smoked			0.91 (0.82; 1.02)			
BMI				0.037		0.243
Normal			Reference		Reference	
Overweight			0.98 (0.90; 1.07)		0.96 (0.88; 1.04)	
Obese			1.09 (1.01; 1.18)		1.06 (0.98; 1.15)	
Alcohol consumption (days/week)				0.393		
None			Reference			
< 1			0.93 (0.85; 1.02)			
1-2			1.03 (0.94; 1.11)			
3-4			0.97 (0.85; 1.12)			
≥ 5			1.05 (0.87; 1.27)			
Physical activity				0.170		0.166
No			Reference		Reference	
Yes			0.95 (0.89; 1.02)		0.95 (0.89; 1.02)	
Chronic condition				0.006		0.007
No			Reference		Reference	
Yes			1.13 (1.04; 1.24)		1.13 (1.03; 1.24)	
Hospitalized due to COVID-19						< 0.001
No					Reference	
Yes					1.24 (1.16; 1.32)	
COVID-19 vaccination (doses)						0.003
3-4					Reference	
1-2					1.08 (1.01; 1.18)	
None					1.23 (1.08; 1.41)	

95%CI: 95CI confidence interval; BMI: body mass index; Model 1: sex, age, education, family income, race/ethnicity, and occupational status; Model 2: Model 1 plus smoking, alcohol consumption, physical activity, and chronic conditions; Model 3: Model 2 plus hospitalization due to COVID-19 and COVID-19 vaccination; PR: prevalence ratio.

Note: Poisson regression models with robust variance. Variables with p-value higher than 0.20 remained in the model.

Participants commonly reported persistent fatigue (56.8%), neurological complications (43.9%), headaches (42.1%), coughing (40.1%), hair loss (33.5%), and loss of smell and taste (30.2%) after being infected with COVID-19. Table 3 shows a more in-depth analysis of the factors associated with each symptom. Female participants were associated with a higher likelihood of experiencing fatigue, neurological complications, headaches, and hair loss than males. Participants receiving more than four minimum wages were less likely to experience neurological complications and hair loss than those receiving up to one minimum wage. Participants who were unemployed and those who were both employed and students had a lower probability of loss of smell and taste than those who were only employed. This probability was also lower for participants who were retired and for those receiving federal financial support. On the other hand, participants who were employed and students, those who were retired, and those with other occupational statuses had a higher likelihood of experiencing a continuous cough than those who were only employed. Race/ethnicity was not associated with long COVID.

**Table 3:** Factors associated with the likelihood of symptom-specific long COVID in adults living in Southern Brazil (N = 1,001).

Characteristics	Fatigue	Neurological complications	Cough	Headache	Loss of smell and taste	Hair loss
	PR (95%CI)	PR (95%CI)	PR (95%CI)	PR (95%CI)	PR (95%CI)	PR (95%CI)
Sex						
Male	Reference	Reference	Reference	Reference	Reference	Reference
Female	1.63 (1.33; 2.01)	1.56 (1.25; 1.94)	1.13 (0.93; 1.37)	1.52 (1.22; 1.90)	1.17 (0.92; 1.50)	4.47 (2.97; 6.73)
Family income (minimum wages)						
< 1	Reference	Reference	Reference	Reference	Reference	Reference
1-2	1.10 (0.80; 1.52)	0.88 (0.68; 1.15)	0.91 (0.64; 1.30)	0.97 (0.70; 1.34)	0.91 (0.61; 1.37)	0.81 (0.59; 1.12)
3-4	1.11 (0.83; 1.49)	0.87 (0.68; 1.12)	0.90 (0.64; 1.26)	1.16 (0.86; 1.57)	0.91 (0.62; 1.35)	0.80 (0.60; 1.06)
> 4	0.87 (0.64; 1.19)	0.71 (0.55; 0.91)	0.80 (0.57; 1.13)	0.84 (0.61; 1.16)	0.72 (0.48; 1.08)	0.60 (0.45; 0.80)
Race/Ethnicity						
White	Reference	Reference	Reference	Reference	Reference	Reference
Black	1.02 (0.69; 1.51)	1.16 (0.88; 1.54)	1.11 (0.79; 1.55)	1.05 (0.79; 1.39)	1.20 (0.80; 1.81)	0.57 (0.32; 1.00)
Mixed-race/*Pardo*	1.09 (0.81; 1.47)	0.82 (0.61; 1.10)	0.96 (0.70; 1.31)	0.89 (0.67; 1.19)	0.85 (0.56; 1.28)	1.18 (0.90; 1.55)
Occupational status						
Employed	Reference	Reference	Reference	Reference	Reference	Reference
Unemployed	0.79 (0.57; 1.08)	1.14 (0.88; 1.47)	1.48 (0.97; 2.26)	0.90 (0.71; 1.13)	0.68 (0.50; 0.93)	1.10 (0.80; 1.53)
Student	0.73 (0.50; 1.05)	0.99 (0.76; 1.28)	1.27 (0.78; 2.06)	0.88 (0.65; 1.20)	0.78 (0.52; 1.18)	1.19 (0.89; 1.58)
Employed and student	0.86 (0.62; 1.20)	1.12 (0.95; 1.33)	1.57 (1.01; 2.43)	0.81 (0.63; 1.06)	0.69 (0.49; 0.97)	1.22 (0.99; 1.50)
Retired	0.49 (0.32; 0.76)	0.71 (0.45; 1.10)	1.81 (1.10; 3.00)	0.79 (0.53; 1.18)	0.73 (0.44; 1.21)	0.79 (0.49; 1.27)
Supported by Federal Government	0.25 (0.09; 0.72)	0.66 (0.22; 1.98)	1.52 (0.57; 4.03)	0.53 (0.16; 1.84)	1.41 (0.83; 2.39)	1.19 (0.59; 2.40)
Other	1.14 (0.71; 1.83)	1.35 (0.90; 2.01)	2.26 (1.35; 3.77)	0.96 (0.65; 1.42)	0.92 (0.56; 1.51)	1.34 (0.86; 2.11)
Smoking						
Never smoked	Reference	Reference	Reference	Reference	Reference	Reference
Former-smoker	0.82 (0.58; 1.16)	0.92 (0.68; 1.24)	1.10 (0.75; 1.59)	1.10 (0.88; 1.37)	1.25 (0.95; 1.65)	0.85 (0.49; 1.46)
Smoker	0.77 (0.57; 1.05)	0.89 (0.69; 1.14)	1.09 (0.79; 1.51)	1.30 (1.01; 1.67)	1.40 (1.02; 1.93)	0.92 (0.59; 1.43)
BMI						
Normal	Reference	Reference	Reference	Reference	Reference	Reference
Overweight	0.83 (0.69; 1.00)	1.03 (0.87; 1.23)	1.13 (0.93; 1.36)	0.96 (0.79; 1.15)	0.97 (0.77; 1.23)	1.01 (0.77; 1.33)
Obese	1.19 (0.98; 1.43)	1.19 (1.01; 1.42)	1.08 (0.88; 1.32)	1.12 (0.94; 1.33)	0.95 (0.74; 1.23)	1.13 (0.86; 1.49)
Alcohol consumption (days/week)						
None	Reference	Reference	Reference	Reference	Reference	Reference
< 1	0.89 (0.73; 1.10)	0.82 (0.68; 0.99)	0.86 (0.70; 1.07)	0.87 (0.73; 1.05)	0.77 (0.58; 1.01)	0.84 (0.51; 1.39)
1-2	0.81 (0.66; 1.00)	0.90 (0.75; 1.09)	1.01 (0.83; 1.23)	0.79 (0.65; 0.96)	1.17 (0.93; 1.48)	0.90 (0.42; 1.95)
3-4	0.86 (0.64; 1.16)	0.89 (0.66; 1.19)	1.02 (0.75; 1.39)	0.75 (0.55; 1.03)	0.75 (0.48; 1.15)	0.98 (0.71; 1.36)
≥ 5	1.14 (0.77; 1.70)	1.29 (0.93; 1.80)	1.05 (0.66; 1.66)	0.98 (0.64; 1.49)	0.85 (0.42; 1.73)	1.03 (0.77; 1.37)
Physical activity						
No	Reference	Reference	Reference	Reference	Reference	Reference
Yes	0.80 (0.68; 0.94)	0.86 (0.74; 0.99)	0.82 (0.69; 0.97)	0.83 (0.71; 0.97)	0.95 (0.77; 1.15)	0.95 (0.75; 1.19)
Hospitalized due to COVID-19						
No	Reference	Reference	Reference	Reference	Reference	Reference
Yes	2.18 (1.68; 2.82)	1.57 (1.28; 1.92)	1.61 (1.21; 2.15)	1.25 (0.90; 1.75)	1.84 (1.36; 2.48)	1.61 (0.98; 2.64)
COVID-19 vaccination (doses)						
3-4	Reference	Reference	Reference	Reference	Reference	Reference
1-2	1.28 (1.06; 1.54)	1.04 (0.87; 1.24)	1.12 (0.91; 1.37)	1.06 (0.87; 1.28)	1.32 (1.03; 1.68)	0.69 (0.22; 2.15)
None	1.12 (0.31; 4.10)	1.46 (0.77; 2.77)	0.71 (0.14; 3.59)	1.71 (1.10; 2.65)	0.91 (0.18; 4.58)	0.67 (0.21; 2.17)
Chronic condition						
No	Reference	Reference	Reference	Reference	Reference	Reference
Yes	1.49 (1.23; 1.81)	1.55 (1.28; 1.91)	1.11 (0.91; 1.35)	1.36 (1.10; 1.67)	1.08 (0.85; 1.38)	0.98 (0.75; 1.27)

95%CI: 95% confidence interval; BMI: body mass index; PR: prevalence ratio.

Note: Poisson regression models with robust variance. Bold values indicate p < 0.05.

Among behavioral characteristics, smoking was associated with an increased likelihood of headaches and loss of smell and taste compared to those who never smoked. Obesity was associated with an increased likelihood of neurological complications than normal weight. Participants who consumed alcoholic beverages < 1 day/week were shown to have a lower likelihood of developing neurological complications, and those who drank alcohol 1-2 days/week were shown to have a lower likelihood of experiencing headaches due to COVID-19. Physical activity was associated with a lower likelihood of fatigue, neurological complications, coughing, and headaches.

Most clinical factors were associated with symptom-specific long COVID. Participants who were hospitalized due to COVID-19 were shown to have a higher likelihood of experiencing fatigue, neurological complications, coughing, and loss of smell and taste. The probability of suffering from fatigue and headaches was higher for participants with incomplete vaccination schedules than for those with complete ones. Lastly, chronic conditions were associated with a higher likelihood of fatigue, neurological complications, and headaches.

## Discussion

Our study described the factors associated with long COVID in adults living in southern Brazil. Three out of four participants developed long COVID after being infected with SARS-CoV-2. Fatigue, neurological complications, headaches, coughing, hair loss, and loss of smell and taste were the most common persistent symptoms experienced after infection. Sociodemographic, behavioral, and COVID-19-related factors - including hospitalization and vaccination - were associated with long COVID. Given the high number of COVID-19 cases in Brazil, public health policies must prioritize strategies to mitigate the forecasted burden of long COVID in the country.

The WHO declared end to the COVID-19 global health emergency on May 5, 2023, primarily due to the increasing vaccination coverage, which reduced the incidence of COVID-19 cases and deaths worldwide ^12^. However, the burden of long COVID is expected to be as high as that of COVID-19 itself, especially in countries considered epicenters of the pandemic ^2^. Previous investigations suggested that 80% of patients who survived COVID-19 reported experiencing persistent symptoms after undergoing the acute phase of the SARS-CoV-2 infection, which corroborates our findings ^1^ ^,^ ^4^. During active infection, people with COVID-19 can experience a wide range of symptoms that are likely to persist after the acute infection stage, including shortness of breath, coughing, loss of smell and taste, and fatigue, as can be observed in our findings. Reducing the burden of long COVID is a global public health priority. Much like in the initial stage of the COVID-19 pandemic, people with long COVID are more likely to suffer from stigma and to need healthcare support. They are at higher risk of complications in multiple organ systems, which can lead to disability ^13^ ^,^ ^14^ ^,^ ^15^ ^,^ ^16^.

Female participants were shown to have a higher likelihood of developing long COVID than males, as observed in previous studies ^1^ ^,^ ^14^ ^,^ ^17^ ^,^ ^18^ ^,^ ^19^. Harmonized data from nine prospective studies, totaling 6,907 adults living in the United Kingdom, showed that women had a 49% higher chance of developing long COVID than men ^18^. The same association was confirmed in a meta-analysis with 120,970 adults averaging 52.3 years of age ^20^. We also observed a higher likelihood of fatigue, neurological complications, hair loss, and headaches in women than in men. Several factors may explain why women are at higher risk of experiencing post-COVID-19 symptoms. Firstly, the fact that psychological stress is more prevalent in women and has been linked to the development of post-COVID-19 symptoms. A previous investigation revealed that individuals with pre-pandemic mental disorders are at higher risk of developing long COVID ^21^. Another factor suggesting that women are more likely to experience persistent COVID-19 symptoms than men is the perception of COVID-19 as a health condition, which was higher in women than men ^22^. Lastly, studies showed that the worsening in anxiety, depression, and sleep disorders due to the COVID-19 pandemic was higher in women than men, and that outbreak-related factors such as social isolation and physical inactivity may exacerbate the risk of post-COVID-19 symptoms in women ^23^ ^,^ ^24^. Similarly, we observed that participants receiving up to one minimum wage and those in unstable occupational positions (i.e., students) were more likely to report persistent neurological symptoms, coughing, and hair loss, which corroborates the potential role of social and economic vulnerability in the burden of long COVID ^25^. Other unmeasured factors may also explain the observed associations of income and occupational status with long COVID, and should be investigated in future studies.

Moreover, physical activity was associated with a lower likelihood of fatigue, neurological complications, coughing, and headaches, corroborating previous findings ^26^ ^,^ ^27^. Since the beginning of the COVID-19 pandemic, several studies have confirmed the beneficial association between physical activity and other COVID-19 outcomes, including infection rate, vaccination effectiveness, hospitalization, and mortality ^28^ ^,^ ^29^. A study analyzed 614 patients aged 56 ± 13 years who were discharged from a tertiary hospital in São Paulo, Brazil, and the authors concluded that participants with long COVID were more likely to be physically inactive at baseline than healthy controls ^26^. A second study observed that adults (n = 477, aged 45 ± 10 years) with long COVID were less active and required more assistance with activities of daily living than in the pre-COVID-19 pandemic ^27^. Physical activity is widely recognized as a non-pharmacological strategy to improve cardiorespiratory fitness, muscular strength, and brain health ^30^ ^,^ ^31^. For example, exercise-induced hormones such as irisin are released through muscle contraction and trigger the production of neurotrophic factors in the brain, which are associated with improved cognitive functions and neuropsychiatric symptoms ^32^. However, physical activity levels were drastically reduced during the COVID-19 pandemic ^24^ ^,^ ^33^. Physical activity must be incorporated into public health policies adapted to populations at risk in order to reduce the burden of long COVID in adults.

Our study is the first to reveal an inverse dose-response relationship between vaccine doses and long COVID in Brazil. An incomplete vaccination schedule was associated with a higher probability of long COVID than a complete schedule. Participants who had not been vaccinated against COVID-19 or received up to two vaccine doses were, respectively, 23% (95%CI: 1.08; 1.41) and 8% (95%CI: 1.01; 1.18) more likely to develop long COVID than those who took three to four doses. A previous community-based study with 28,356 adults living in the United Kingdom and aged from 18-69 found similar results: as the number of vaccine doses increased, the likelihood of long COVID decreased ^34^. Additionally, a French study with 910 adults showed that immunization reduced the number and burden of long COVID symptoms ^35^.

Based on our findings and other studies, vaccination against COVID-19 seems to decrease the prevalence of long COVID, since it reduces the likelihood of persistent symptoms in those who are already symptomatic when vaccinated, preventing breakthrough infections and reducing the risk of the disease in the first place, as well as the transmission of the virus after infection ^36^. The dose-response relationship observed in this study and in previous ones suggests that vaccination could benefit individuals with long COVID who may experience immune system dysregulation. Vaccination could “reset” autoimmune processes, although its long-term effects are still uncertain. Additionally, antibody responses strengthened by vaccination could help to destroy any residual viral reservoir in the body. Another potential mechanism of long COVID that immunization could help fight is the persistence of viral antigens modifying the immune response months after infection. Thus, our findings reinforce the need of universal access to COVID-19 vaccination in order to reduce the burden of long COVID.

Moreover, we observed that participants who had been hospitalized due to COVID-19 were more likely to develop long COVID than those who had not been hospitalized, but other studies on this topic have provided conflicting results. Previous investigations suggested that the prevalence of long COVID is similar between individuals who have been hospitalized and those who have not ^37^ ^,^ ^38^. Another meta-analysis also showed no differences between hospitalization status ^21^. In contrast, a meta-analysis showed that previous hospitalization or intensive care unit (ICU) admission was associated with a high risk of long COVID, supporting our findings ^36^. Factors such as the differences between healthcare systems across countries may explain the heterogeneity in this association. For example, COVID-19-related symptoms such as shortness of breath are better controlled in individuals who are hospitalized than in non-hospitalized individuals. However, longer waiting times for hospitalization due to healthcare system disruption, which was common during the most acute stage of the COVID-19 pandemic, especially in low- and middle-income countries, could lead to a more severe development of these symptoms.

Our study found that individuals with chronic conditions such as hypertension and diabetes were more likely to experience long COVID. This supports previous outcomes from systematic reviews and meta-analyses ^1^ ^,^ ^20^ ^,^ ^36^ ^,^ ^37^. In Brazil, people living with chronic conditions were prioritized in social distancing policies and vaccination campaigns due to the higher risk of COVID-19-related hospitalization and mortality ^39^ ^,^ ^40^. Our findings reinforce the need for continuous surveillance and public health policies to reduce the burden of COVID-19-related complications, including long COVID, in people with chronic conditions.

In contrast, we also found that occasional alcohol consumption was associated with a lower prevalence of neurological complications and headaches after SARS-CoV-2 infection. This is likely because cardiometabolic conditions such as obesity and diabetes can create a pro-inflammatory condition that promotes the persistence of inflammation and related symptoms for longer, thereby increasing the risk of long COVID. On the other hand, low alcohol consumption decreases the body’s inflammatory status, which could explain the protective association between occasional alcohol consumption and long COVID ^41^. However, one should note the uncertainty of the evidence about the proinflammatory and anti-inflammatory effects of low-dose alcohol consumption. Further investigation is needed to improve the understanding of the factors influencing the development of long COVID.

The main strength of this study is the novelty of its research topic. This is the first study to estimate the number of people living with long COVID in Brazil, an epicenter of the COVID-19 pandemic, and the factors associated with this condition in free-living adults. However, the study also has some limitations that must be acknowledged. First, COVID-19 infection was self-reported by participants, as the local ethics committee did not allow in-person research activities during the baseline assessments. We also did not investigate the presence of long COVID in asymptomatic patients. However, previous meta-analysis showed that the asymptomatic SARS-CoV-2 infection was not associated with an increased risk of symptoms, daily activity impairment, hospitalization, or death ^42^. Similarly, we identified cases of long COVID considering COVID-19-related symptoms that persisted for at least three months, as previously recommended by the WHO ^43^. Our findings might underestimate the true prevalence of long COVID - it is possible to acknowledge this by observing studies that used different cut-offs (e.g., four weeks) ^44^ ^,^ ^45^. Another limitation is that the cross-sectional design, used in this study, does not allow causal inference between the analyzed factors and long COVID, although other cohort studies have corroborated some of our findings ^34^ ^,^ ^36^. The third limitation is the fact that we used an online questionnaire to conduct our research, which may have resulted in sampling bias and prevented us from reaching a representative sample. However, our study design was feasible to analyze the unprecedented impact of the COVID-19 pandemic on the population’s health.

In conclusion, our findings reveal that three out of every four adults in southern Brazil have long COVID. Sociodemographic and behavioral factors were associated with a higher likelihood of this condition. The prevalence of long COVID was lower in vaccinated individuals than in unvaccinated ones. This condition affect different organ systems, including neurological complications and fatigue. Public health policies adapted to high-risk groups, such as constant vaccination campaigns, are urgently needed to reduce the burden of long COVID.
